# Serum Galectin-9 and Galectin-3-Binding Protein in Acute Dengue Virus Infection

**DOI:** 10.3390/ijms17060832

**Published:** 2016-05-27

**Authors:** Kuan-Ting Liu, Yao-Hua Liu, Yen-Hsu Chen, Chun-Yu Lin, Chung-Hao Huang, Meng-Chi Yen, Po-Lin Kuo

**Affiliations:** 1 Graduate Institute of Clinical Medicine, College of Medicine, Kaohsiung Medical University, Kaohsiung 807, Taiwan; kuantingliu7@gmail.com; 2 Department of Emergency Medicine, Kaohsiung Medical University Hospital, Kaohsiung Medical University, Kaohsiung 807, Taiwan; lau0619@yahoo.com.tw; 3 School of Medicine, College of Medicine, Kaohsiung Medical University, Kaohsiung 807, Taiwan; infchen@gmail.com (Y.-H.C.); infectionman@gmail.com (C.-Y.L.); locusthao@gmail.com (C.-H.H.); 4 Division of Infectious Diseases, Department of Internal Medicine, Kaohsiung Medical University Hospital, Kaohsiung 807, Taiwan; 5 Department of Biological Science and Technology, College of Biological Science and Technology, National Chiao Tung University, Hsinchu 300, Taiwan; 6 Institute of Medical Science and Technology, National Sun Yat-Sen University, Kaohsiung 804, Taiwan

**Keywords:** galectin, glycoprotein, serum, dengue, galectin-9, galectin-3 binding protein, adult, fever

## Abstract

Dengue fever is a serious threat for public health and induces various inflammatory cytokines and mediators, including galectins and glycoproteins. Diverse immune responses and immunological pathways are induced in different phases of dengue fever progression. However, the status of serum galectins and glycoproteins is not fully determined. The aim of this study was to investigate the serum concentration and potential interaction of soluble galectin-1, galectin-3, galectin-9, galectin-3 binding protein (galectin-3BP), glycoprotein 130 (gp130), and E-, L-, and P-selectin in patients with dengue fever in acute febrile phase. In this study, 317 febrile patients (187 dengue patients, 150 non-dengue patients that included 48 patients with bacterial infection and 102 patients with other febrile illness) who presented to the emergency department and 20 healthy controls were enrolled. Our results showed the levels of galectin-9 and galectin-3BP were significantly higher in dengue patients than those in healthy controls. Lower serum levels of galectin-1, galectin-3, and E-, L-, and P-selectin in dengue patients were detected compared to bacteria-infected patients, but not to healthy controls. In addition, strong correlation between galectin-9 and galectin-3BP was observed in dengue patients. In summary, our study suggested galectin-9 and galectin-3BP might be critical inflammatory mediators in acute dengue virus infection.

## 1. Introduction

Dengue fever is a mosquito-borne viral disease and is caused by dengue virus (type I–IV) [1]. A statistical report from World Health Organization indicates the incidence of dengue fever has been significantly rising in the past 50 years [2]. Especially in recent decades, the impact of dengue has increased geographically in tropical and subtropical areas including Taiwan [3,4]. More than 30,000 dengue patients were reported in Kaohsiung from 2014 to 2015 according to the statistics of the Taiwan National Infectious Disease Statistics System [5]. The outbreak dengue virus is mainly genotype I and serotype I in Kaohsiung [6]. It is a serious threat in southern Taiwan and other tropical areas.

After mosquito bite, initial dengue virus infection takes place in keratinocytes and dendritic cells [7]. Following infection, dengue virus affects various types of cells, including endothelial cells, liver cells, monocytes, and lymphocytes [8]. Elevated levels of cytokines and chemokines such as tumor necrosis factor-α, macrophage migration inhibitory factor, monocyte chemoattractant protein-1, interleukin (IL)-1β, and IL-8 are detected in serum of patients and some molecules correlated with disease severity and clinical outcome [9,10,11,12,13]. Because infection of dengue virus triggers innate immune responses, some inflammatory mediators such as galectins associated with dengue infection are also induced [14]. Galectins are a family of mammalian lectins and are released under stress condition such as infection [15]. The roles of galectin-1 and galectin-9 in dengue infection have been determined [16,17]. In addition, the serum levels of soluble adhesion molecules E-, P- and L-selectin, which are transmembrane glycoproteins, are altered by dengue infection [18,19,20]. These studies suggest galectins and glycoproteins are important factors for regulating immune responses during dengue infection. However, these studies did not fully determine serum concentration and interaction of all molecules in each specific phase of dengue infection.

Clinically, onset of dengue is observed as an acute febrile phase on Days 1–3, a critical (plasma leak) phase of illness on Days 4–6 and convalescence phase on Days 7–10. Expect for dengue fever, severe dengue virus infection results in thrombocytopenia, plasma leakage and shock (dengue hemorrhagic fever/dengue shock syndrome (DHF/DSS)) [21,22]. In the present study, the dengue patients in acute febrile phase were enrolled. Sera of adult fever patients with dengue infection, non-dengue illness, and healthy donors were collected. The non-dengue illness group was further divided into bacterial infection group and other febrile illness group. The levels of galectins and glycoproteins in all groups were analyzed by Luminex assay.

## 2. Results

### 2.1. Laboratory Feature

In this study, a total of 317 febrile patients including 187 dengue patients, 150 non-dengue patients which included 48 patients with bacterial infection and 102 patients with other febrile illness (OFI)), and 20 healthy controls were studied between November 2013 and November 2015 (Table 1). All patients diagnosed with dengue were further confirmed by the Taiwan Centers for Disease Control. The count of white blood cell and platelet in dengue patients were significantly lower than that in total non-dengue patients. In addition, the level of hemoglobin, and C-reactive protein in dengue patients was significantly different from that in total non-dengue patients (Table 2). Because bacterial infection showed unique symptoms and there were 48 bacteria-infected patients in OFI group, we further determined whether there were different levels of these laboratory features among all groups. In the bacterial infection group, higher levels of white blood cell count, C-reactive protein, blood urea nitrogen, creatinine and lower level of hemoglobin was detected compared to the dengue group. In addition, higher levels of white blood cell count, platelet count, and C-reactive protein was observed in the OFI group.

### 2.2. Serum Level of Galectins and Glycoproteins

**Figure 1:**
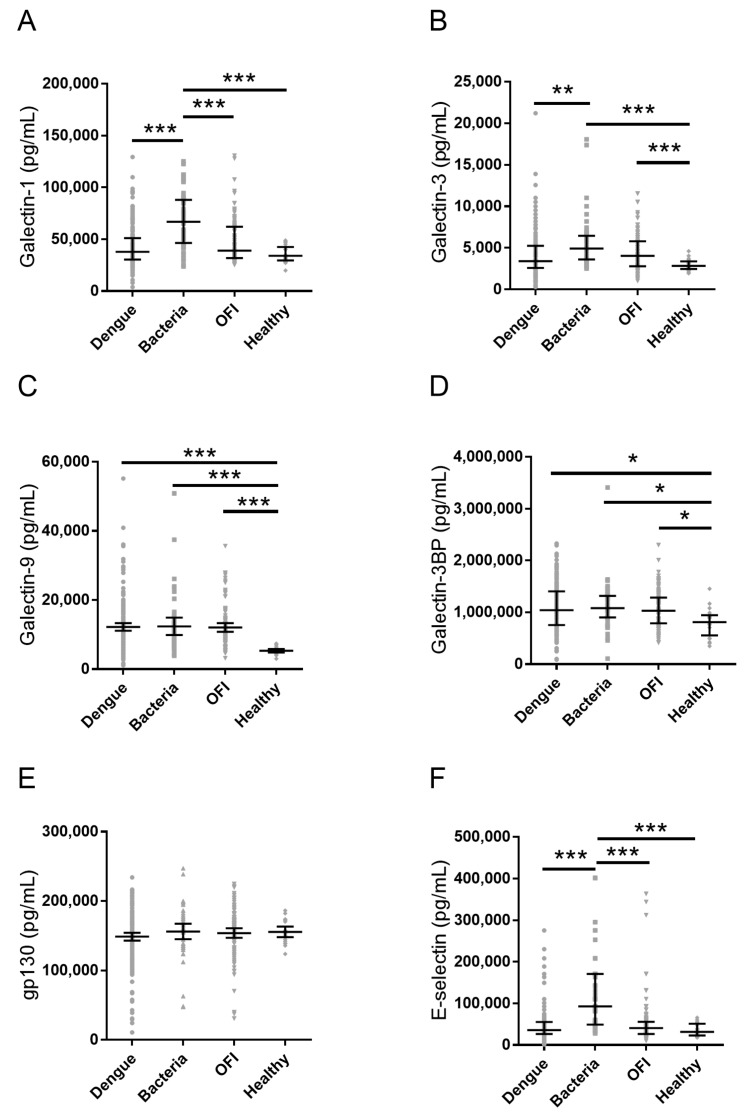

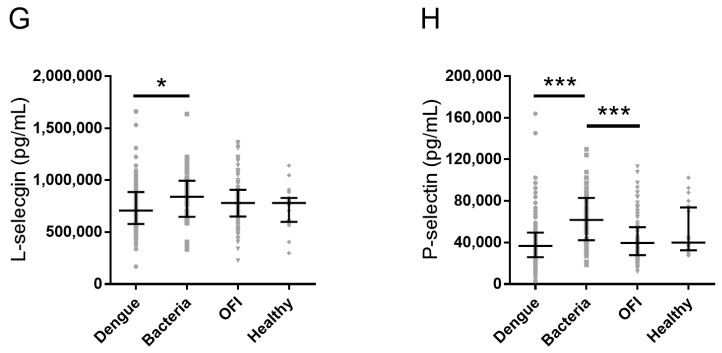
The levels of galectins and glycoproteins. Aligned dot plot showing the serum concentration of: (**A**) galectin-1; (**B**) galectin-3; (**C**) galectin-9; (**D**) galectin-3BP; (**E**) gp130; (**F**) E-selectin; (**G**) L-selectin; and (**H**) P-selectin. Data represent median with interquartile range; * *p* < 0.05; ** *p* < 0.01; *** *p* < 0.0001.

The serum concentration of soluble galectins (galectin-1, galectin-3, and galectin-9), and glycoproteins (galectin-3-binding protein (galectin-3BP)), glycoprotein 130 (gp130), E-selectin, L-selectin, and P-selectin) were determined by Luminex assay. The result is shown in Table 3 and Figure 1 as median and interquartile range.

#### 2.2.1. Galectin-1

Higher level of galectin-1 was found in the bacterial infection group than in other groups (37,953 pg/mL (30,429–51,153) (dengue) *vs*. 66,963 pg/mL (48,842–87,145) (bacterial infection) *vs*. 39,055 pg/mL (32,068–61,693) (OFI) *vs*. 34,063 pg/mL (30,399–42,519) (healthy controls)). However, there was no significant difference between dengue patients and healthy controls.

#### 2.2.2. Galectin-3

In the bacterial infection group, the level of galectin-3 was higher compared to dengue and healthy control groups. In addition, the level of galectin-3 in the OFI group was higher than that in the healthy control group, but no significant difference between dengue group and healthy controls was observed (3411 pg/mL (2630–5,262) (dengue) *vs*. 4056 pg/mL (2807–5736) (bacterial infection) *vs*. 4930 pg/mL (3631–6327) (bacterial infection) *vs*. 2837 pg/mL (2505–3303) (healthy controls)).

#### 2.2.3. Galectin-9

The level of galectin-9 in healthy controls was significantly lower than in other groups (10,287 pg/mL (7197–14,868) (dengue) *vs*. 9852 pg/mL (7806–13,932) (bacterial infection) *vs*. 10,515 pg/mL (7743–14,161) (OFI) *vs*. 5061 pg/mL (4691–6018) (healthy controls)). No significant difference of galectin-9 level was detected in all patient groups.

#### 2.2.4. Galectin-3BP

The expression pattern of galectin-3BP was similar with galectin-9. The lowest level of galectin-3BP was observed in healthy controls among all groups (1,040,481 pg/mL (754,569–1,405,380) (dengue) *vs*. 1,082,591 pg/mL (902,473–1,316,118) (bacterial infection) *vs*. 1,032,948 pg/mL (789,621–1,278,950) (OFI) *vs*. 881,495 pg/mL (657,756–937,917) (healthy controls)).

#### 2.2.5. GP130

No significant difference of soluble gp130 level was observed among all groups (155,355 pg/mL (143,980–170,740) (dengue) *vs*. 159,957 pg/mL (135,942–175,034) (bacterial infection) *vs*. 159,957 pg/mL (139,633–172,465) (OFI) *vs*. 125,847 pg/mL (129,963–173,181) (healthy controls)).

#### 2.2.6. E-selectin

In bacterial infection group, the level of E-selectin was significantly higher than other groups (35,721 pg/mL (26,379–55,092) (dengue) *vs*. 92,652 pg/mL (49,982–170,730) (bacterial infection) *vs*. 40,680 pg/mL (26,379–54,507) (OFI) *vs*. 31,434 pg/mL (23,038–47,933) (healthy controls)).

#### 2.2.7. L-selectin

Except for the dengue group and bacterial infection group, there was no significant difference among other groups (707,409 pg/mL (579,360–863,175) (dengue) *vs*. 841,656 pg/mL (665,972–982,706) (bacterial infection) *vs*. 780,363 pg/mL (654,533–907,590) (OFI) *vs*. 780,363 pg/mL (611,704–823,264) (healthy controls)).

#### 2.2.8. P-selectin

The level of P-selectin in bacterial infection group was higher than in dengue and OFI groups, but it was not significantly different from healthy controls (36,672 pg/mL (25,932–49,662) (dengue patients) *vs*. 61,674 pg/mL (43,148–81,041) (bacterial infection) *vs*. 39,562 pg/mL (28,278–54,156) (OFI) *vs*. 39,900 pg/mL (33,270–71,412) (healthy controls)).

### 2.3. Correlation between Cell Counts and Each Molecule

We determined whether galectins and glycoproteins correlated with number of white blood cells or platelets in the dengue group. However, no significant correlation was observed among both parameters and galectins and glycoproteins in the dengue group (Table 4) and bacterial infection and OFI groups (data not shown). Because galectin–glycoprotein interaction is observed in various conditions, the correlation between each soluble molecule was also determined. In Table 5, moderate to strong correlation was observed between galectin-1 and galectin-3; galectin-1 and galectin-9; galectin-3 and galectin-9; galectin-3 and E-selectin; galectin-9 and galectin-3BP; and E-selectin and P-selectin in dengue patients. The results of bacterial infection and OFI groups are shown in Table 6 and Table 7.

## 3. Discussion

There are no specific therapeutic agents for dengue [22]. Investigation of host immune responses and inflammatory mediators that are induced during dengue virus infection may be beneficial to determine regulatory mechanism of anti-dengue immune responses and develop vaccines or specific drug against dengue infection. In this study, the comparison among dengue patients who were in acute febrile phase, patients with bacterial infection, patients with other febrile illness and healthy people for three soluble galectins and five soluble glycoproteins were performed. The level of galectin-9 and galectin-3BP in dengue patients was higher than in healthy controls. In addition, high expression of galectin-9 and galectin-3BP was also observed in bacterial infection and OFI groups. The highest level of galectin-1, galectin-3, and E-selectin was detected in the bacterial infection group.

In clinical settings, white blood cell counts, C-reactive protein and procalcitonin could be used to facilitate the diagnosis of bacterial infection [23,24]. Low total white blood cell counts, neutrophils and monocyte counts, and platelet counts are clinical characteristics for diagnosis of acute dengue illness [25,26]. Classic characteristics of enrolled patients with dengue and bacterial infection are observed in Table 2. In addition, the OFI group also showed higher white blood cell counts, platelet counts and C-reactive protein level than did the dengue group.

Galectin-1 has been demonstrated to control pathogens infection through inhibiting microbial activity and inducing apoptosis in host cell [27]. A recent study indicated galectin-1 shows inhibitory effect on infection of dengue virus type-1 [17]. As shown in Figure 1A, bacterial infection induced the highest level of galectin-1 among all groups. The galectin-1 level in dengue was not significantly different from galectin-1 level of healthy controls. The result suggests serum galectin-1 is not induced in the acute febrile phase. Although it is reported dengue virus type I is the dominating type of dengue virus in this outbreak in Kaohsiung [6], the serotype and genotype of dengue virus of each enrolled dengue patient was not determined in this study. Therefore, the role of galectin-1 needs to be further investigated under infection of dengue virus type I in clinic.

Galectin-3 is crucial in innate immunity for bacterial and viral infection [28,29]. It is reported to bind to lipopolysaccharide on various bacteria species and suppress LPS-induced inflammation [30]. In Figure 1B, the levels of galectin-3 in bacterial infection and OFI groups, but not in dengue group, was higher than those in healthy controls group. The result suggests galectin-3 was less affected by dengue infection in the acute febrile phase compared to other fever illness.

Very low plasma level of galectin-9 is detected in healthy people [31]. Elevation plasma level of galectin-9 is detected after dengue virus infection and it is associated with disease severity [16]. The level of galectin-9 is correlated with dengue virus titer [16]. In critical phase and recovery phase of dengue fever, the level of galectin-9 is significantly associated with IL-8, interferon-γ inducible protein 10, vascular endothelial growth factor (VEGF) and negatively correlated with monocyte percentage [16]. In addition, the level of serum or plasma galectin-9 was observed during various types of viral infection, such as human immunodeficiency virus, hepatitis B virus, hepatitis C virus and influenza [32]. Galectin-9 serves as a negative regulator of anti-viral immune responses by inducing apoptosis of CD8^+^ T cell and expanding regulatory T cells during several types of viral infections [32]. It is currently unclear whether dengue virus affects CD8^+^ T cells and regulatory T cells. In the present study, the levels of galectin-9 in three groups of patients were significantly higher than in healthy controls. Galectin-9 level was not significantly associated with WBC count and platelet count. It suggests galectin-9 might be associated with induction of different types of cytokines and immune cells in acute febrile phase.

Galectin-3BP, also known as Mac-2 binding protein, was found in response to *in vitro* dengue virus infection [33]. High serum level of galectin-3BP was detected in human immunodeficiency virus-, hantavirus- and hepatitis C virus-infected individuals [34,35]. Previous reports indicate elevated RNA level of galectin-3BP is detected in various types of dengue virus-infected cells including muscle cells, monocytes, B cells, and human umbilical vein endothelial cells [36,37]. It has been demonstrated as one of the antiviral type I interferon inducible genes [36]. In addition, galectin-3BP interacts with galectin-1, galectin-3, galectin-7 and E-selectin [38,39]. Although the biological functions of galectin-3BP are not fully understood, this evidence suggests that it is an important regulator for anti-virus and anti-bacteria immune responses. Our results showed the level of galectin-3BP in healthy donors was significantly lower than that in all of the patient groups. We suppose galectin-3BP may play an important role in generating immune responses against dengue virus in acute febrile phase.

It is interesting to note strong correlation between galectin-9 and galectin-3BP, but no significant correlation between galectin-9 and galectin-3BP in the bacterial infection group (Table 5 and Table 6). However, the function of both molecules was unclear in acute febrile phase of dengue. A previous report hypothesizes that galectin-9 plays a role in regulating migration and adhesion of monocyte on endothelial cells [16]. Since galectin-9 and galectin-3BP are reported to be induced from dengue virus infected-endothelial cells [36,40], we assume that both molecules regulate the interaction between monocytes and endothelial cells in acute febrile phase of dengue. The detail mechanism of galectin-9 and galectin-3BP needs to be further determined.

IL-6 plays an important role in immune responses against bacterial and viral infection [41,42]. GP130 is a component in IL-6 receptor complex and is induced under human hepatitis C virus infection [43]. Surprisingly, our results revealed serum concentration of soluble gp130 was not induced in dengue, bacterial, and OFI patients although gp130 level significantly correlates with some molecules in each group. It may imply gp130 is only induced by other types of pathogens.

Serum levels of glycoprotein soluble E-, L- and P-selectin are associated with infectious diseases. E-selectin is a marker of activated endothelial cell, and soluble E-selectin is induced in various infectious diseases including sepsis and dengue virus infection [20,44,45]. A report described the levels of soluble E-selectin in dengue fever and dengue hemorrhagic fever patients were lower than in healthy controls [19]. Our results showed the level of E-selectin in dengue patients was not significantly different from healthy controls. L-selectin is a lymphocyte-associated molecule and P-selectin is a platelet-associated molecule respectively [18]. Although white blood cell counts and platelet counts significantly decreased in dengue patients compared to healthy controls and other patients, the levels of L-selectin and P-selectin in dengue patients was not different compared to healthy controls in this study. In addition, the levels of L- and P-selectin did not correlate with white blood cell counts and platelet counts. A study indicates that mean plasma concentration of L- and P-selectin in dengue virus 2-infected patients is not significantly higher than in healthy controls [18]. However, Paris and colleagues indicated higher concentrations of L-selectin in serum in dengue fever and dengue hemorrhagic fever patients compared to healthy people [19]. The difference of E-, L- and P-selectin among these studies needs to be further investigated in the future.

In this study, the serum was collected when patients presented to the emergency department. We did not collect serum at the following time points. Therefore, the expression pattern of galectins and glycoproteins in each dengue phase and different levels of severity could not be evaluated. Furthermore, our study indicated strong correlation between serum concentrations of galectin-9 and galectin-3BP in febrile dengue patients. However, there were not sufficient experimental evidences to investigate whether galectin-9 was associated with expansion of regulatory T cells, galectin-3BP activated T cells and NK cells because the number of macrophages, neutrophils, NK cells was not measured and immunophenotyping assays were not performed in this study.

## 4. Materials and Methods

### 4.1. Sample Collection

Adult fever patients (tympanic temperature > 38.3 °C, age > 18 years old) who presented to the emergency department of Kaohsiung Medical University Hospital (Kaohsiung, Taiwan) with fever from September 2014 to October 2015 were eligible for the study. Blood from all patients and health donors were obtained after they agreed and signed informed consent. The use of the serum samples in this study was approved by the Institutional Review Board of Kaohsiung Medical University Hospital (IRB Number: KMUH-IRB-20120287). Ten milliliters of blood from fever patients were drawn in serum separation tubes and then stored in aliquots in −80 °C. Because dengue is a notifiable disease in Taiwan, sera from patients with suspected dengue infection should be sent to the Taiwan Centers for Disease Control where final confirmation of the diagnosis is made through Dengue Ag NS1 Strip (Bio-Rad, Maines-la-Couqette, France). Some of dengue patients were further confirmed by RT-PCR. The criteria of bacterial infection were according to positive blood culture results. Sera of dengue patients, non-dengue patients and healthy donors were used for the following Luminex assay (Millipore, St Charles, MO, USA).

### 4.2. Quantification of Serum Molecules

The serum molecules were quantitated by using an 8-plex Human Magnetic Luminex Screening Assay (R&D Systems, Minneapolis, MN, USA). The concentrations of soluble galectin-1, galectin-3, galectin-9, galectin-3BP, gp130, E-selectin, L-selectin, and P-selectin in serum were determined according to the manufacturer’s instructions. Data were acquired on Luminex xMAP technology (Millipore, St Charles, MO, USA). For concentration calculation, a five-parameter logistic curve fit curve method was performed using the Milliplex Analyst Software (Viagene Tech, Carlisle, MA, USA).

### 4.3. Statistical Analysis

Differences between two independent groups were analyzed with Mann–Whitney U test (for calculating the difference between dengue and non-dengue groups in Table 2). Comparisons between three or four groups were done with Kruskal–Wallis test, followed by Dunn’s multiple comparison test. The results were shown as the median and the interquartile range. All calculation and graphing were carried out using GraphPad Prism version 5.03 (GraphPad Software, San Diego, CA, USA).

## 5. Conclusions

In acute febrile phase, galectin-9 and galectin-3BP were induced in dengue patients compared to healthy controls. Detailed immunological regulations need to be investigated in further studies. The levels of galectin-1, galectin-3, L-selectin and P-selectin in dengue patients were different from those in patients with bacterial infection, but it was not different from those in healthy controls. Furthermore, strong correlation between galectin-9 and galectin-3BP was observed in acute dengue infection. Our results revealed that both molecules might be important inflammatory mediators in acute dengue virus infection.

## Figures and Tables

**Table 1:** Enrolled patients and healthy controls.

Characteristic	Dengue Patients	Non-Dengue Patients			Healthy Controls
		Total	Bacterial	OFI	
Age	50.56 ± 18.31	54.61 ± 20.37	65.2 ± 15.1	49.62 ± 20.9	38.05 ± 7.89
Gender (Male/Total)	90/187	72/150	27/48	45/102	8/20

Age was shown: mean ± SD.

**Table 2:** Laboratory features.

Laboratory Features	Dengue Patients (*n* = 187)	Non-Dengue Patients		
		Total (*n* = 150)	Bacterial (*n* = 48)	OFI (*n* = 102)
WBC (× 1000/μL)	4.335 (2.93–6.21)	8.55 (6.4–12.5) *	11.38 (7.33–14.78) *	8.25 (5.8–11.18) *
Platelet (× 1000/μL)	148 (100–200.25)	192 (136–247) *	169 (124.75–240.25)	189 (149–249) *
HB (mg/dL)	14.1 (12.9–15.3)	13.5 (11.5–14.6) *	11.85 (10.33–13.53) *	13.5 (12.1–14.9)
CRP (mg/L)	7.38 (3.75–14.7)	14.99 (8.36–114.14) *	108.4 (32.05–186.3) *	15.92 (4.38–50.38) *
BUN (mg/dL)	11.2 (8.9–15.3)	12.3 (9.92–19.6)	16 (11.98–24) *	11.8 (9.25-16.08)
CR (mg/dL)	0.85 (0.7–1.11)	0.88 (0.78–1.18)	1.08 (0.82–1.66) *	0.88 (0.76–1.11)
Glu (mg/dL)	124 (107–154)	119 (107.75–165.75)	151 (112–195)	118 (104–151.25)

Data are shown as median (interquartile range); Symbol: * significant difference between dengue group (*p* < 0.01); CRP: C-reactive protein; WBC: White blood cell count; Hb: Hemoglobin; BUN: Blood urea nitrogen; Glu: Glucose; Cr: Creatinine. Some data was undetectable in some patients.

**Table 3:** Serum galectins and glycoproteins.

Galectins and Glycoproteins	Dengue Patients	Non-Dengue Patients	Healthy Controls
Galectin-1	37,953 (30,429–51,153)	48,963 (34,046–70401) *	34,063 (30,399–42,519)
Galectin-3	3,411 (2,630–5,262)	4,236 (2,995–5,991) *	2,837 (2,505–3,303) *
Galectin-9	10,287 (7,197–14,868)	10,287 (7,535–13,932)	5,061 (4,691–6,018) *
Galectin-3BP	1,040,781 (754,569–1,405,380)	1,060,806 (804,315–1,294,738)	811,495 (657,756–937,917) *
gp130	152,847 (129,963–173,781)	159,957 (138,903–173261)	155,355 (143,980–170,740)
E-selectin	35,721 (26,379–55,092)	46,326 (31,539–84,960) *	31,434 (23,038–47,933)
L-selectin	707,409 (579,360–863,175)	800,214 (654,533–907,590) *	780,363 (611,704–823,264)
P-selectin	36,672 (25,932–49,662)	43,611 (30,837–67,257) *	39,990 (33,270–71,412)

Data are shown as median (interquartile range); Symbol: * significant difference between dengue group (*p* < 0.05).

**Table 4:** Correlation between galectins and glycoproteins, and white blood cell (WBC) and platelet counts in dengue patients.

Galectins and Glycoproteins	WBC Count		Platelet Count	
	*r*	*p* Value	*r*	*p* Value
Galectin-1	0.042	0.572	−0.012	0.868
Galectin-3	0.041	0.577	0.063	0.392
Galectin-9	−0.046	0.526	−0.086	0.242
Galectin-3BP	−0.084	0.252	−0.068	0.353
gp130	0.095	0.194	−0.014	0.846
E-selectin	0.134	0.068	0.068	0.359
L-selectin	−0.054	0.461	−0.138	0.061
P-selectin	0.095	0.198	0.135	0.066

*r*: Spearman’s correlation coefficient.

**Table 5:** Correlation between galectins and glycoproteins in dengue patients.

Galectins and Glycoproteins	Galectin-1	Galectin-3	Galectin-9	Galectin-3BP	gp130	E-Selectin	L-Selectin
Galectin-1							
Galectin-3	0.508 *						
Galectin-9	0.478 *	0.499 *					
Galectin-3BP	0.204	0.326	0.609 *				
gp130	0.341	0.396 *	0.338 *	0.166			
E-Selectin	0.382 *	0.464 *	0.103	0.134	0.400 *		
L-Selectin	0.090	0.236	0.323 *	0.298 *	0.224	0.152	
P-selectin	0.270	0.304 *	−0.107	−0.061	0.245	0.432 *	0.130

Spearman’s correlation coefficient is shown; * *p* < 0.001.

**Table 6:** Correlation between galectins and glycoproteins in bacteria-infected patients.

Galectins and Glycoproteins	Galectin-1	Galectin-3	Galectin-9	Galectin-3BP	gp130	E-Selectin	L-Selectin
Galectin-1							
Galectin-3	0.615 *						
Galectin-9	0.731 *	0.656 *					
Galectin-3BP	0.160	0.147	0.294				
gp130	0.485	0.400	0.466	0.048			
E-Selectin	0.061	0.263	0.100	0.052	0.390		
L-Selectin	−0.071	0.199	0.058	0.236	−0.051	0.125	
P-selectin	0.227	0.367	0.076	−0.013	0.532 *	0.414	0.076

Spearman’s correlation coefficient is shown; * *p* < 0.001.

**Table 7:** Correlation between galectins and glycoproteins in OFI patients.

Galectins and Glycoprotein	Galectin-1	Galectin-3	Galectin-9	Galectin-3BP	gp130	E-Selectin	L-Selectin
Galectin-1							
Galectin-3	0.371 *						
Galectin-9	0.386 *	0.287					
Galectin-3BP	0.152	0.154	0.465 *				
gp130	0.410 *	0.500 *	0.282	0.198			
E-Selectin	0.434 *	0.460 *	0.162	0.221	0.480 *		
L-Selectin	0.268	0.186	0.104	0.108	0.224	0.429 *	
P-selectin	0.276	0.046	−0.165	−0.139	0.166	0.361 *	0.256

Spearman’s correlation coefficient is shown; * *p* < 0.001.
